# Synergistic Effects of Lignin Fiber and Sodium Sulfate on Mechanical Properties and Micro-Structure of Cement-Stabilized Soil

**DOI:** 10.3390/ma18173929

**Published:** 2025-08-22

**Authors:** Liang Wang, Binbin Na, Wenhua Chen

**Affiliations:** 1 Institute of Disaster Prevention Engineering, Zhejiang Institute of Hydraulics and Estuary (Zhejiang Institute of Marine Planning and Design), Hangzhou 310020, China; 2 School of Civil Engineering and Architecture, Zhejiang Sci-Tech University, Hangzhou 310018, China

**Keywords:** stabilized soil, lignin fiber, sodium sulfate, synergistic effect, mechanical properties, micro-structure

## Abstract

This study aims to develop environmentally friendly soil-stabilization materials by investigating the synergistic enhancement mechanism of industrial by-product lignin fibers (LFs) and sodium sulfate (Na_2_SO_4_) on the mechanical and micro-structural properties of cement-stabilized soil. A systematic evaluation was conducted through unconfined compressive strength (UCS), splitting tensile strength, and capillary water absorption tests, supplemented by microscopic analyses including XRD and SEM. The results indicate that the optimal synergistic effect occurs at 1.0% LF and 0.10% Na_2_SO_4_, which increases UCS and splitting tensile strength by 9.23% and 18.37%, respectively, compared to cement-stabilized soil. Meanwhile, early strength development is accelerated. Microscopically, LF physically bridges soil particles, forming aggregates, reducing porosity, and enhancing cohesion. Chemically, Na_2_SO_4_ acts as an activator, accelerating cement hydration and stimulating pozzolanic reactions to form calcium aluminosilicate hydrate and gypsum, which fill pores and densify the matrix. The synergistic mechanism lies in Na_2_SO_4_ enhancing the interaction between the LFs and clay minerals through ion exchange, facilitating the formation of a stable spatial network structure that inhibits particle sliding and crack propagation. This technology offers substantial sustainability benefits by utilizing paper-making waste LF and low-cost Na_2_SO_4_ to improve soil strength, toughness, and impermeability.

## 1. Introduction

Soil stabilization is a crucial engineering technique for improving the mechanical properties (e.g., strength and stiffness) and durability (e.g., water and erosion resistance) of soil [1,2], with wide applications in roadbed construction and foundation treatment [3,4]. Although traditional stabilizers such as cement, lime, and fly ash are effective [5,6], their production and use are associated with high carbon emissions, resource depletion, and environmental burdens. These drawbacks hinder their alignment with the growing demand for sustainable development. By partially replacing cement with industrial waste, it is expected that the carbon dioxide emissions per cubic meter of stable soil can be reduced by 0.42 kg. Therefore, developing efficient, low-carbon, and cost-effective alternative stabilization materials and technologies holds substantial engineering and environmental value.

In recent years, bio-based materials have emerged as a promising research direction in soil stabilization due to their renewability and environmental compatibility. Among them, lignin fiber (LF)—a major by-product of the paper-making industry—stands out for its abundance, low cost, and favorable physico-chemical properties [7]. Owing to its fibrous structure and adhesive nature, LF enhances soil cohesion and reduces erosion by forming a reinforcing network that binds soil particles [8,9]. Studies [10,11] have shown that LF can effectively inhibit crack propagation in soil, attributed to its unique morphology and surface functional groups. The stabilization mechanism of LF is primarily based on physical fiber bridging, pore-filling effects, and inter-facial interactions with soil particles [12,13]. These characteristics underscore the potential of LF as an effective, sustainable soil stabilizer and reinforcing agent. In parallel, chemical additives also play a crucial role in improving the performance of stabilized soil. Sodium sulfate (Na_2_SO_4_), a low-cost inorganic salt, enhances soil stability through mechanisms such as ion exchange and mineral crystallization [14,15]. In sulfate-rich environments, Na_2_SO_4_ reacts with calcium ions to generate gypsum, which can effectively fill soil pores and improve compaction [16,17]. A similar stabilization mechanism has been reported in the use of phosphogypsum, where sulfate-based additives promote the formation of stable hydration products [18,19], thereby enhancing material strength and durability; however, it is important to note that excessive sulfate content may lead to volumetric expansion or the formation of ettringite, potentially compromising structural integrity [20,21]. The issue of using sulfate additives to stabilize the durability of soil deserves attention [22]. Therefore, precise control of sulfate dosage is essential to optimize performance while avoiding adverse effects.

This study aims to explore the synergistic interaction between lignin fiber and sodium sulfate in cement-stabilized soils, a combination not yet systematically investigated in the literature. Although both LF and Na_2_SO_4_ have demonstrated distinct advantages as individual additives in soil stabilization, the current research still exhibits notable limitations and knowledge gaps. Existing studies on LF predominantly focus on its adsorption capacity or its role as a component in composite materials, with insufficient attention paid to its physical reinforcement mechanisms in soil, particularly its synergistic interactions with traditional cementitious materials [23,24]. In contrast, research on Na_2_SO_4_ has mainly emphasized its chemical activation effects, especially its influence on the hydration behavior and stability of phosphogypsum-based materials. So far, the maximum potential of Na_2_SO_4_ in enhancing the performance of stabilized soil has not yet been fully exploited; moreover, investigations into the combined application of LF and Na_2_SO_4_ in soil stabilization remain scarce. Specifically, the potential synergistic mechanisms—such as the interaction between the fiber-reinforced network formed by LF and the hydration products induced by Na_2_SO_4_—have not been thoroughly elucidated. Furthermore, there is a lack of systematic studies addressing the optimization of LF and Na_2_SO_4_ proportions and their combined effects on the overall performance of cement-stabilized soil, including parameters such as mechanical strength, toughness, water resistance, and micro-structural characteristics. The novelty lies in revealing the dual physical–chemical reinforcement mechanism using industrial by-products, offering an eco-friendly alternative to conventional soil stabilizers.

To overcome the limitations of traditional stabilizers and fully utilize the advantages of industrial by-products such as LF and inexpensive chemicals such as Na_2_SO_4_, this study proposes and deeply explores the synergistic enhancement mechanism of LF and Na_2_SO_4_ in cement-stabilized soil. The aim of this study is to systematically evaluate the synergistic effect through macroscopic mechanical and physical performance tests such as unconfined compressive strength, splitting tensile strength, and capillary water absorption rate, quantifying the improvement effect of different LF contents combined with Na_2_SO_4_ on the performance of cement–lime-stabilized soil; to reveal the microscopic mechanism by comprehensively using microscopic analysis techniques such as X-ray diffraction, scanning electron microscopy, energy dispersive spectroscopy, and back-scattered electron imaging, characterizing the influence of LF and Na_2_SO_4_ on the micro-structure and pore evolution of the stabilized soil; and to clarify the essence of their physical and chemical synergistic effects. This study not only provides a scientific basis and practical solutions for developing high-performance and environmentally friendly soil-stabilization technologies using industrial waste LF and low-cost Na_2_SO_4_, it also reveals the synergistic mechanisms and has significant engineering application value and environmental benefits.

## 2. Experimental Program

### 2.1. Raw Materials

**Figure 1:**
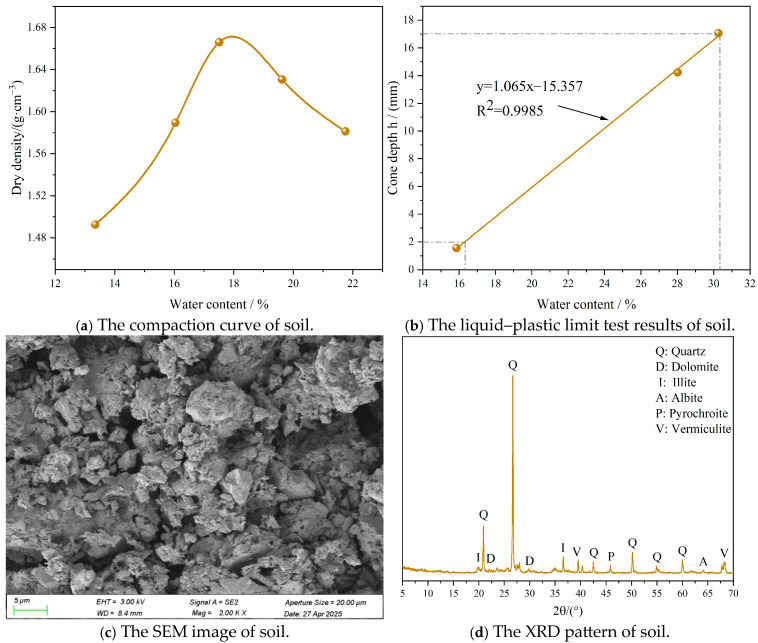
Basic physical properties of soil.

The soil samples analyzed in this study were obtained from construction waste produced during foundation pit excavation in Jiande County, Hangzhou, China. The initial moisture content was 28.6%, and the samples exhibited high toughness and a relatively hard texture. After preliminary crushing with a mixer (Jianglin Instrument, Hebi, China), no coarse aggregates were observed. The maximum dry density and optimum moisture content were determined via standard light compaction tests [25,26]. Five moisture content levels were selected for testing, and the resulting dry density–moisture content relationship is illustrated in Figure 1a. The maximum dry density and optimum moisture content were found to be 1.67 g/cm^3^ and 17.94%, respectively. The liquid limit and plastic limit were determined using a combined liquid-and-plastic-limit apparatus (Yixuan Testing, Cangzhou, China) [27]. The correlation between moisture content and cone penetration depth is shown in Figure 1b. The liquid limit and plastic limit were 30.4% and 16.3%, respectively, yielding a plasticity index of 14.1. A summary of the physical properties is provided in Table 1. The micro-structure of the soil was examined using a modern scanning electron microscope (Quanta, FEI, Hillsboro, OR, USA), as shown in Figure 1c. Mineral particles were predominantly sheet-like, with particle contacts characterized by edge–edge, face–face, and edge–face interactions. Inter-particle voids were also observed. As seen in Figure 1d, X-ray diffraction (XRD-7000, Shimadzu Corporation, Kyoto, Japan) analysis of the untreated soil sample revealed that the dominant minerals were quartz, albite, dolomite, pyrochroite, illite, and vermiculite.

Portland cement and lime were selected as the primary binders for soil stabilization. The cement used was 32.5-grade ordinary Portland cement, supplied by Qianjiang Cement Factory (Hangzhou, China). The lime used was provided by Guoxun Group Chemical Reagents Co., Ltd. (Tianjin, China). It appeared as a white, granular powder. LF, an industrial by-product of the paper-making industry, was incorporated as a natural additive. It is light brown in color and emits a plant-like aroma. Na_2_SO_4_, used as a chemical reagent, was of analytical grade and obtained from Tianjin Zhiyuan Chemical Reagent Co., Ltd. (Tianjin, China).

### 2.2. Mix Design and Specimen Preparations

In this study, cement and lime were used as curing agents. Based on preliminary optimization, a cement-to-lime ratio of 3:1 was found to yield optimal stabilization performance [28,29]. To investigate the synergistic enhancement effects of LFs and Na_2_SO_4_ on cement-stabilized soil, the total dosage of the curing agent was fixed at 7% of the dry soil mass, and the water content was set at the previously determined optimum value of 17.94%. LFs were added at three dosage levels: 0.5%, 1.0%, and 1.5% by mass. Additionally, with the LF content fixed at 1.0%, Na_2_SO_4_ was introduced at three concentrations: 0.05%, 0.10%, and 0.15%. The specific experimental design scheme is shown in Table 2.

The test soil was first oven-dried at 105 °C for over 24 h, then ground and passed through a 4.75 mm sieve for subsequent use. The sieved soil was thoroughly mixed with the designated amounts of curing agents and water according to the experimental design. Mixing was performed using a cement mortar mixer (Mengyuan Testing Instruments, Cangzhou, China) to ensure uniformity, and sample preparation was carried out immediately to minimize moisture loss. Specimens were fabricated using a single-step compaction molding method with a cylindrical steel mold (φ50 mm × H50 mm) (Luxin Testing Instruments, Cangzhou, China). After compaction, the specimens were left undisturbed for 2 min, demolded using a release device, sealed with plastic wrap, and placed in a curing chamber under standard conditions (temperature 20 ± 2 °C, relative humidity ≥ 95%) until the designated curing age.

### 2.3. Test Methods

To evaluate the feasibility of incorporating LF and Na_2_SO_4_ as additives in cement-stabilized soil, a series of experiments was conducted to assess both mechanical and micro-structural properties. The experimental program included unconfined compressive strength (UCS) tests at 3, 7, and 28 days, indirect tensile strength (splitting) test at 28 days, capillary water absorption test at 28 days, as well as micro-structural analyses using X-ray diffraction (XRD), scanning electron microscopy (SEM), and back-scattered surface sweep analysis (Quanta, FEI, Hillsboro, OR, USA).

#### 2.3.1. Unconfined Compressive Strength Tests

Cylindrical specimens (φ50 mm × H50 mm) were subjected to unconfined compressive strength (UCS) tests after standard curing for 3, 7, and 28 days. The tests were performed using a 5-ton universal testing machine (Lian Gong Testing, Jinan, China), with the axial loading rate maintained at 1 mm/min [30]. For each curing age, three parallel specimens were tested, and the average value was reported as the UCS. Additionally, the elastic modulus of the cement-stabilized soil was determined from the slope of the linear ascending portion of the stress–strain curve obtained during the UCS test.

#### 2.3.2. Indirect Tensile Strength Test

The indirect tensile strength of the cement-stabilized soil was evaluated using the Brazilian splitting test on cylindrical specimens (φ50 mm × H50 mm). Each specimen was placed horizontally in the loading fixture of the testing machine (Lian Gong Testing, Jinan, China), with 3-millimeter-thick wooden pads positioned at both contact edges to ensure even load distribution. The loading was applied at a constant rate of 1 mm/min until the specimen fractured along its diameter [31].

#### 2.3.3. Capillary Water Absorption Test

To investigate the capillary pore structure of cement-stabilized soil, a capillary water absorption test was performed [32,33]. Specimens cured for 28 days were oven-dried to a constant mass over a period of two days. The lateral surfaces of each specimen were sealed with epoxy resin to ensure unidirectional water ingress through the bottom surface only. Prior to testing, the initial mass of each sample was recorded. During the test, the water level was maintained at 5 mm above the bottom surface. At predetermined time intervals, the specimens were removed, and any surface moisture was gently wiped off with a towel. The mass of water absorbed was measured using a high-precision horizontal electronic balance.

#### 2.3.4. X-Ray Diffraction Tests

Following the unconfined compressive strength test, the freshly crushed specimens were dried, ground, and sieved to obtain fine powders with a particle size of approximately 10 μm for X-ray diffraction (XRD) analysis. The XRD tests were conducted using a D8 Advance diffractometer operated at 40 kV. The scanning was performed at a rate of 2°/min over the range of 5° to 70° [34].

#### 2.3.5. Scanning Electron Microscopy Tests

To elucidate the micro-structure and hydration products of cement-stabilized soil, scanning electron microscopy (SEM) and energy dispersive spectroscopy (EDS) (Quanta, FEI, Hillsboro, OR, USA) analyses were performed to investigate the influence of LF and Na_2_SO_4_ on the performance. Following the unconfined compressive strength test, a specimen approximately 1 cm^3^ in size was selected for SEM analysis. To terminate hydration, the specimen was immersed in anhydrous ethanol and then dried at a constant temperature of 40 °C for 24 h. Prior to imaging, the sample was coated with gold using an automatic magnetron ion sputtering device. SEM observations were then carried out to examine the cross-sectional morphology and identify hydration products.

## 3. Results

### 3.1. Analysis of Traditional Cement-Stabilized Soil

**Figure 2:**
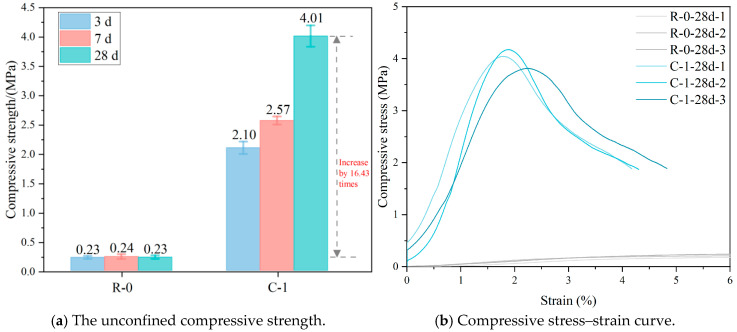
The compressive properties of soil and cement-stabilized soil.

The compressive performance of compacted in situ soil and cement-stabilized soil is presented in Figure 2. The unconfined compressive strength of the compacted in situ soil (R-0) remained consistently low, ranging from 0.23 to 0.24 MPa at 3, 7, and 28 days of curing, indicating negligible cementation and confirming the lack of hydraulic properties. In contrast, the cement-stabilized soil (C-1) exhibited a significant strength increase, reaching 2.10 MPa at 3 days, approximately 8 times higher than R-0, primarily due to the rapid hydration of cement and the early formation of calcium silicate hydrate (C-S-H) gels. At 28 days, the strength increased to 4.01 MPa, which is 16.43 times that of R-0. This substantial improvement highlights the synergistic effect of the cement–lime dual cementation system. While cement hydration contributed to early strength development, the subsequent activation of lime facilitated long-term pozzolanic reactions between Ca(OH)_2_ and clay minerals [35,36], resulting in the formation of additional calcium aluminosilicate hydrate (C-A-S-H) gels that further densified the matrix and reduced porosity. The compressive stress–strain curves of the 28-day-cured specimens demonstrate the significant impact of the cement–lime stabilizer on the mechanical behavior of the soil. The R-0 exhibited typical ductile failure in all repeated tests, characterized by low inter-particle bonding and gradual micro-crack propagation. In contrast, the C-1 displayed brittle failure behavior, with a noticeably steeper initial elastic slope, indicative of matrix densification due to binder addition. The post-peak response of C-1 showed gradual softening rather than abrupt failure, suggesting quasi-ductile behavior arising from the progressive coalescence of micro-cracks within the C-S-H/C-A-S-H gel network formed during hydration.

**Figure 3:**
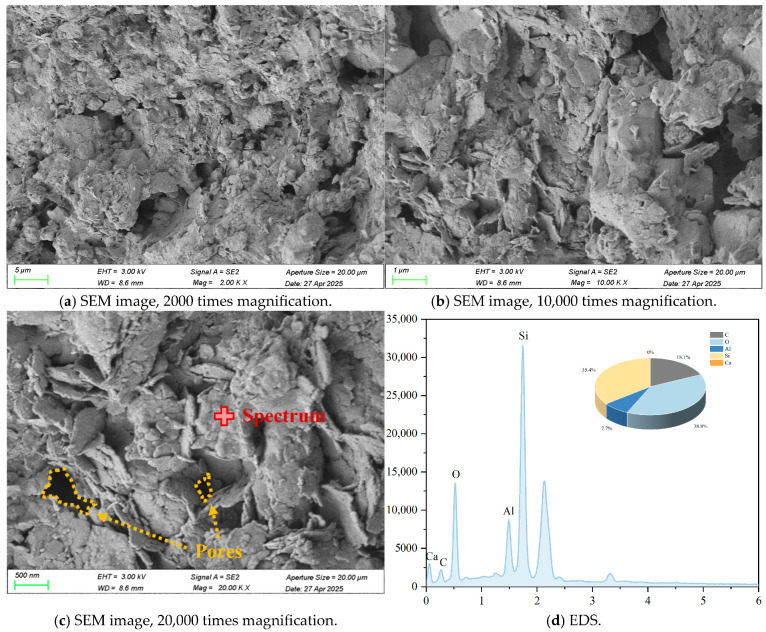
Typical SEM images and EDS elemental analysis of R-0 samples at 28 days.

**Figure 4:**
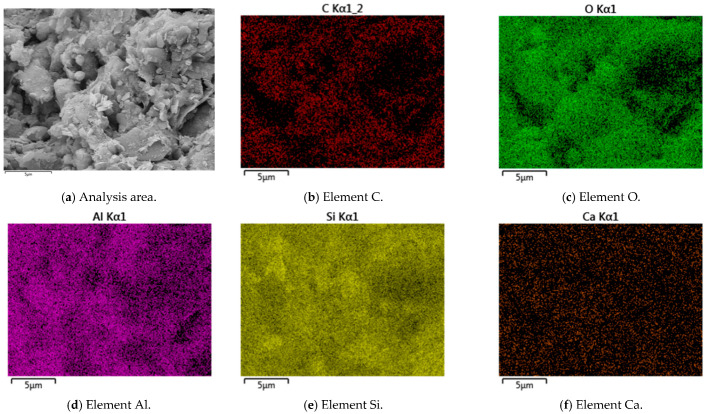
Back-scattering surface scanning analysis of R-0 samples at 28 days.

**Figure 5:**
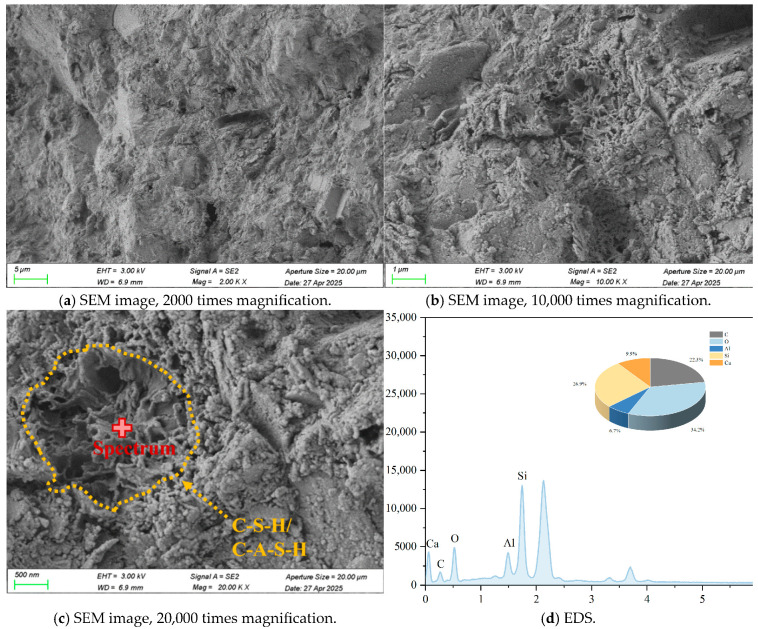
Typical SEM images and EDS elemental analysis of C-1 samples at 28 days.

**Figure 6:**
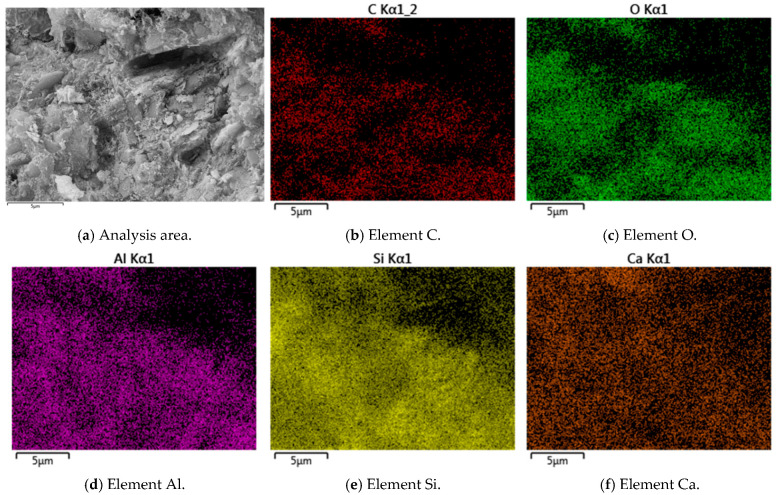
Back-scattering surface scanning analysis of C-1 samples at 28 days.

Typical SEM images, EDS elemental, and back-scattering surface scanning analysis of R-0 and C-1 samples at 28 days are shown in Figure 3, Figure 4, Figure 5 and Figure 6. Micro-structural analysis of the R-0 at 28 days of curing reveals fundamental limitations contributing to its poor mechanical performance. SEM imaging shows a loosely packed particulate structure dominated by face-to-edge contacts between platy clay particles, with no evidence of cementitious gels or crystalline bonding phases at particle interfaces, accounting for the lack of cohesive strength development over time. Elemental mapping via EDS further confirms chemical inertness, with no calcium-enriched zones detected, thereby ruling out any pozzolanic reactions. In contrast, cement–lime stabilization induces substantial changes in the soil micro-structure. While R-0 remains a loose and porous assemblage, the C-1 exhibits a significantly denser matrix, with hydration products such as C-S-H and C-A-S-H gels coating clay particles and forming a continuous bonding network [37]. These hydration products act as binders and fillers, effectively bridging particles and reducing macro-pores. The transformation from a granular, geologically loose matrix in R-0 to a cohesive, viscous-like solid in C-1 is attributed to cement hydration. The resulting pore blockage increases matrix density, which directly contributes to the observed improvement in mechanical strength.

### 3.2. The Unconfined Compression Performance

**Figure 7:**
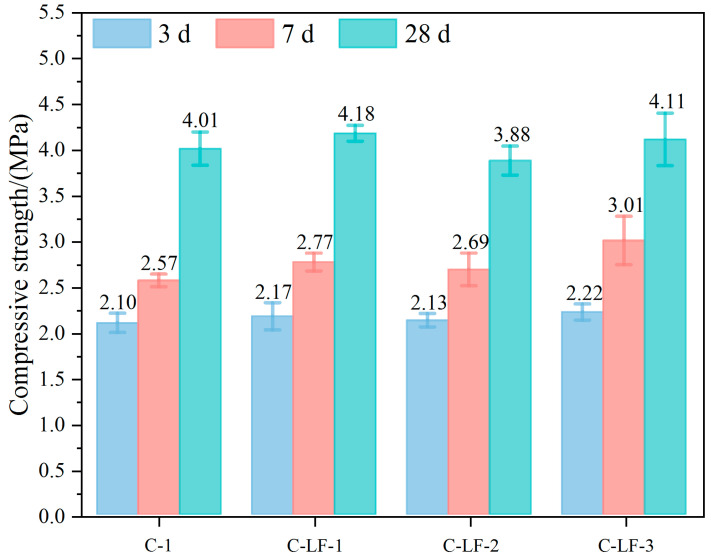
The unconfined compressive strength of cement-stabilized soil with LF.

As shown in Figure 7, the incorporation of LF into cement-stabilized soil leads to a slight increase in UCS, although the overall impact remains limited. At lower dosages, the dispersed LFs fill micro-cracks and enhance matrix integrity, contributing to a modest strength improvement. This enhancement is primarily attributed to the crack-bridging effect of the fibers and their role in reinforcing internal cohesion [38]. However, with increasing LF content, the UCS does not exhibit a proportional rise and eventually plateaus or slightly declines; this results from fiber agglomeration at the higher contents, which disrupts matrix uniformity and impedes cement hydration. Moreover, weak inter-facial bonding between the LFs and hydration products causes local debonding under load [39], diminishing the reinforcing effect.

**Figure 8:**
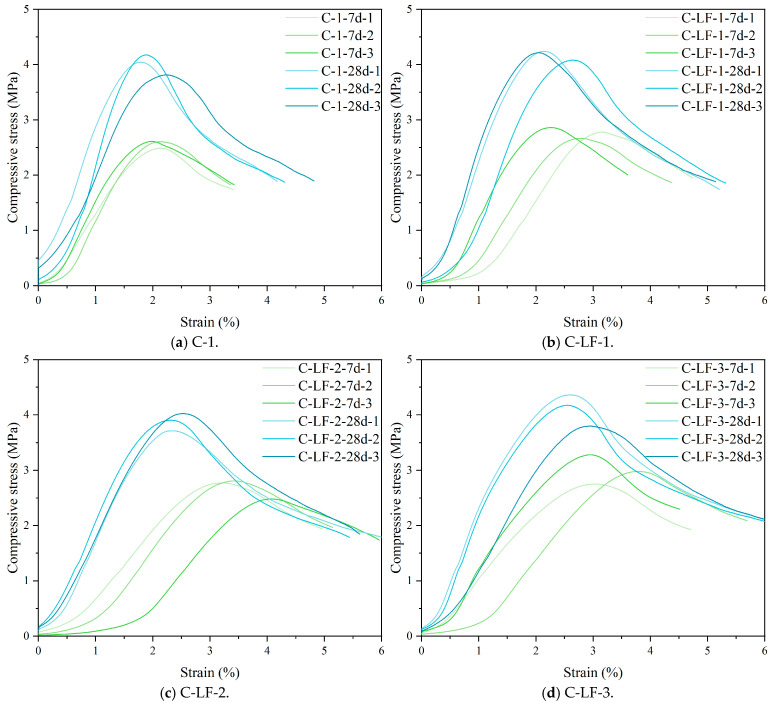
The compressive stress–strain curves of cement-stabilized soil with LF.

Figure 8 presents the compressive stress–strain curves of LF-modified, cement-stabilized soil. The addition of LF significantly improves the ductility of the material. At 0.5% LF content, the C-LF-1 specimen demonstrates greater ductility compared to the C-1, attributed to the effective bridging of micro-cracks by well-dispersed fibers. With LF content increased to 1.0–1.5%, crack resistance is further enhanced. Overall, while LFs contribute to improved structural stability through enhanced ductility and crack resistance, their effect on compressive strength remains relatively modest.

**Figure 9:**
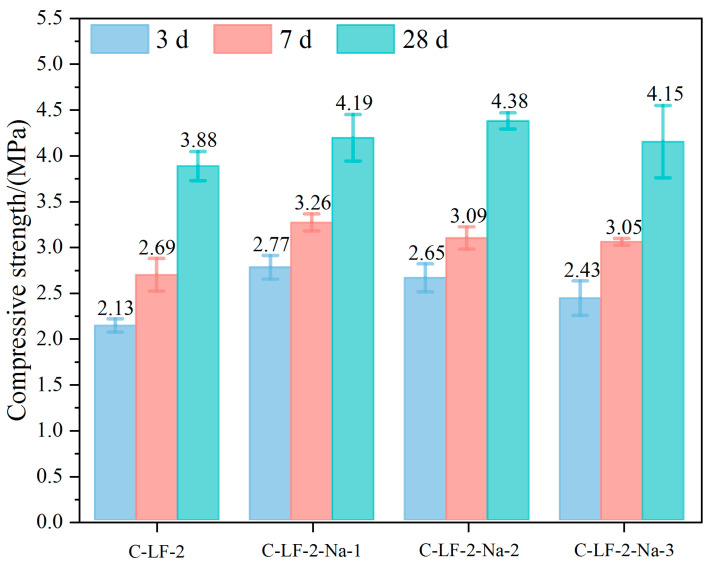
The unconfined compressive strength of cement-stabilized soil with LF and Na_2_SO_4_.

When the strength enhancement effect of LF on cement-stabilized soil is limited, the addition of an appropriate amount of Na_2_SO_4_ produces a synergistic strengthening effect. As shown in Figure 9, the unconfined compressive strength increases significantly when the Na_2_SO_4_ content rises from 0.05% to 0.10%. This improvement is mainly attributed to the Na_2_SO_4_ ability to promote cement hydration and activate pozzolanic reactions of certain mineral components in the soil, leading to the formation of additional C-S-H and C-A-S-H gels that enhance matrix density and strength [40]. However, further increasing the Na_2_SO_4_ content to 0.15% results in a strength reduction, likely due to excessive ion concentration inhibiting cement hydration and promoting the overproduction of by-products such as gypsum or calcium sulfoaluminate [41]. These effects can cause internal stress concentration or increased porosity, ultimately compromising the mechanical integrity of the stabilized soil.

**Figure 10:**
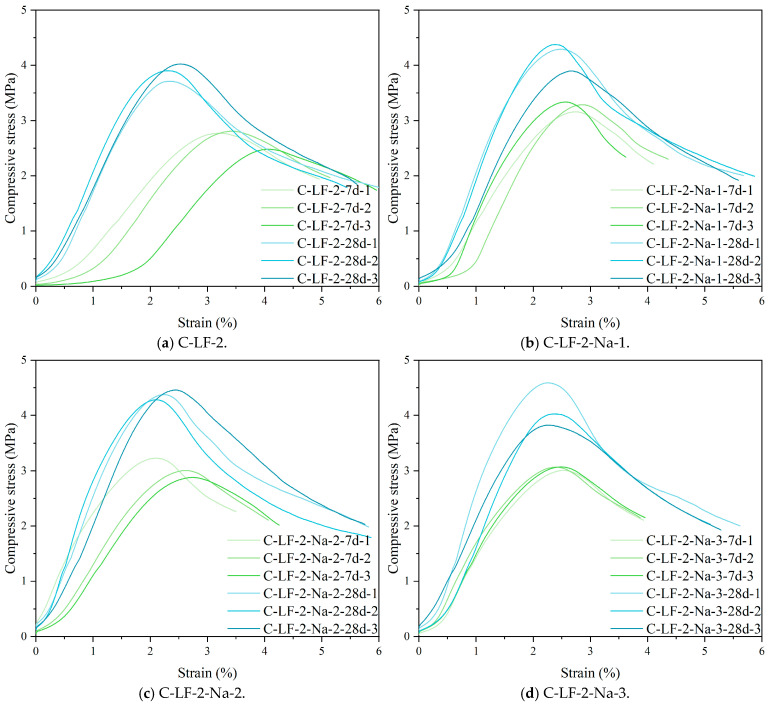
The compressive stress–strain curves of cement-stabilized soil with LF and Na_2_SO_4_.

The compressive stress–strain curves of cement-stabilized soil incorporating LF and Na_2_SO_4_ are presented in Figure 10. As shown, increasing the Na_2_SO_4_ content leads to a gradual reduction in the ductility of the stabilized soil, evidenced by a sharper post-peak stress drop and a more brittle failure mode. This behavior is primarily attributed to the promotion of cement hydration and the pozzolanic reaction of active minerals in the soil by Na_2_SO_4_, which results in a denser matrix structure [42,43,44]. However, the enhanced compactness simultaneously reduces the capacity of internal micro-cracks to buffer and expand under load, thereby limiting the ability to undergo plastic deformation.

**Figure 11:**
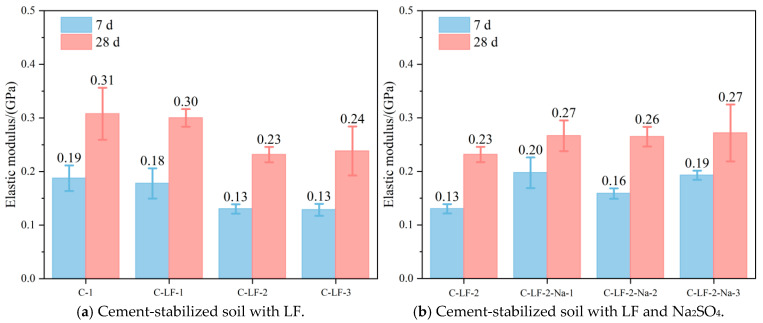
The elastic modulus of cement-stabilized soil.

The elastic modulus of the cement-stabilized soil is presented in Figure 11. As shown, the incorporation of LF slightly decreases the elastic modulus, whereas Na_2_SO_4_ exerts a moderate enhancing effect. The reduction caused by LFs is primarily due to their lower stiffness relative to the cementitious matrix, which introduces more compliant phases into the system and reduces its overall rigidity [45]. Conversely, Na_2_SO_4_ promotes the formation of additional cementitious products, such as C-S-H and C-A-S-H, by accelerating cement hydration and stimulating pozzolanic reactions. This leads to a denser and more rigid micro-structure, thereby slightly increasing the elastic modulus.

### 3.3. Splitting Tensile Strength

**Figure 12:**
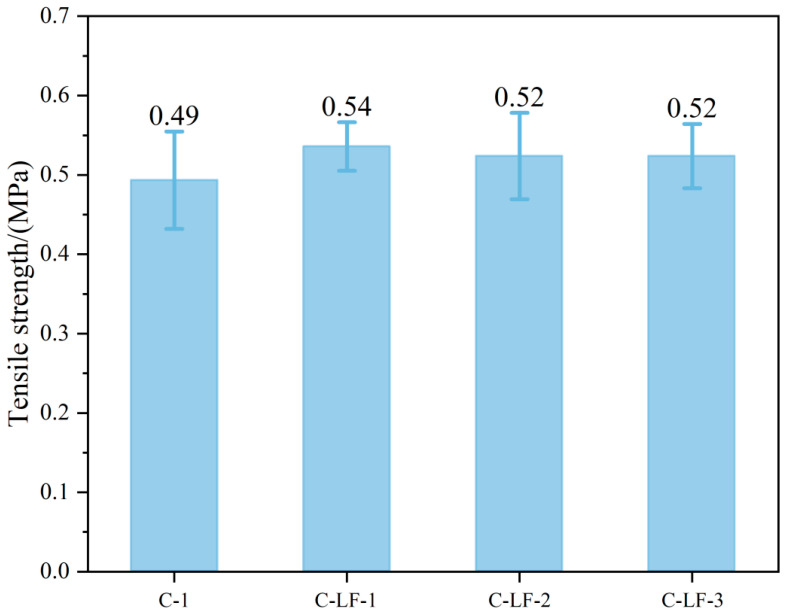
The tensile strength of cement-stabilized soil with LF.

The splitting tensile strength of cement-stabilized soil with LF is shown in Figure 12. Compared to C-1, the tensile strength of C-LF-1, C-LF-2, and C-LF-3 increased by 10.20%, 6.12%, and 6.12%, respectively, indicating that LF incorporation exerts a moderate reinforcing effect. This enhancement is primarily attributed to the bridging and crack-arresting effects of the fibers. LFs form a more uniform three-dimensional network within the matrix. During crack initiation and propagation, the fibers transfer tensile stress across crack surfaces, thereby delaying crack growth and improving tensile resistance. Furthermore, the surface roughness of the fibers enhances inter-facial bonding with the matrix, promoting effective stress transfer [46]. However, due to the inherently low tensile strength of LF, the overall reinforcement remains modest, resulting in only slight improvements in splitting tensile strength.

**Figure 13:**
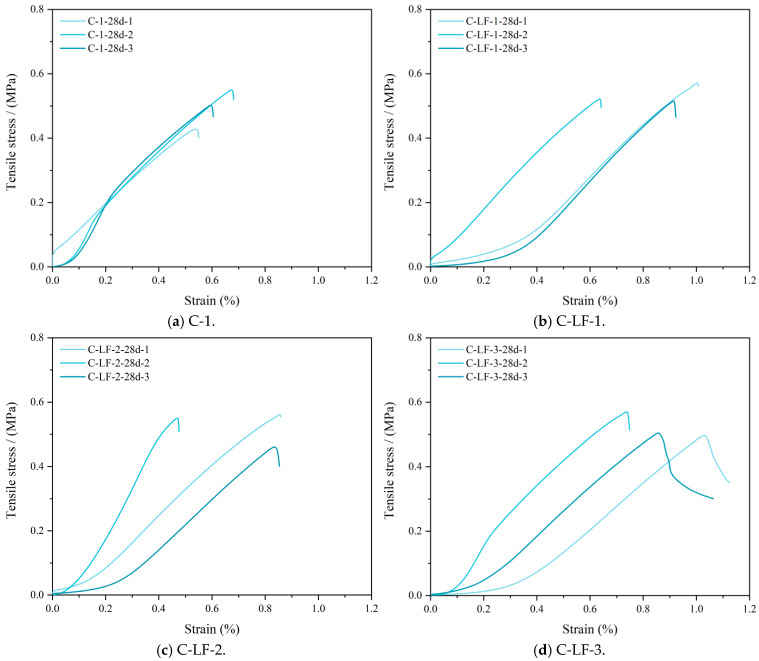
The tensile stress–strain curves of cement-stabilized soil with LF.

The tensile stress–strain curves of cement-stabilized soil with LF are presented in Figure 13. As the LF content increases, the splitting tensile deformation capacity of the stabilized soil is noticeably enhanced, primarily due to the reinforcing effect of the fibers. LFs possess moderate tensile strength and favorable deformation compatibility. Upon crack formation, the fibers bridge the crack surfaces, absorbing part of the tensile stress and delaying crack propagation [47]. This bridging mechanism improves the toughness and plastic deformation capacity. Additionally, fiber pull-out during the failure process dissipates energy, allowing the stabilized soil to accommodate greater strain and reducing its tendency toward brittle failure. Consequently, the inclusion of LF contributes to improved crack resistance and enhanced deformation stability.

**Figure 14:**
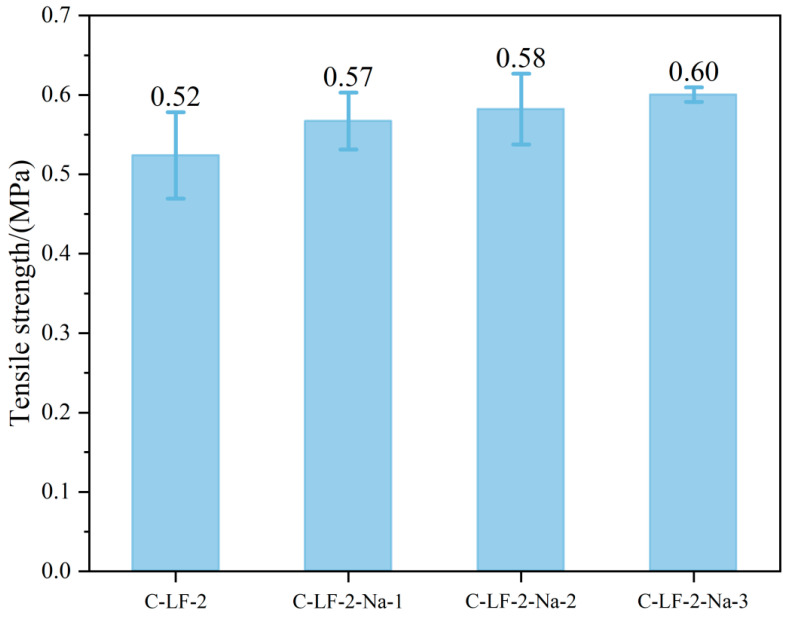
The tensile strength of cement-stabilized soil with LF and Na_2_SO_4_.

The splitting tensile strength of cement-stabilized soil incorporating LF and Na_2_SO_4_ is shown in Figure 14. Compared to C-LF-2, the tensile strength of C-LF-2-Na-1, C-LF-2-Na-2, and C-LF-2-Na-3 increased by 9.62%, 11.54%, and 15.38%, respectively. This enhancement is attributed to the synergistic effect between Na_2_SO_4_ and LF. Acting as a chemical activator, Na_2_SO_4_ accelerates the hydration reaction and promotes the formation of additional C-S-H gels, resulting in a denser matrix and stronger inter-facial bonding. The improved micro-structure enhances the adhesion between fibers and the matrix, effectively limiting fiber slippage and enabling more efficient stress transfer through fiber bridging [48]. As a result, the overall tensile strength and crack resistance of the stabilized soil are significantly improved.

**Figure 15:**
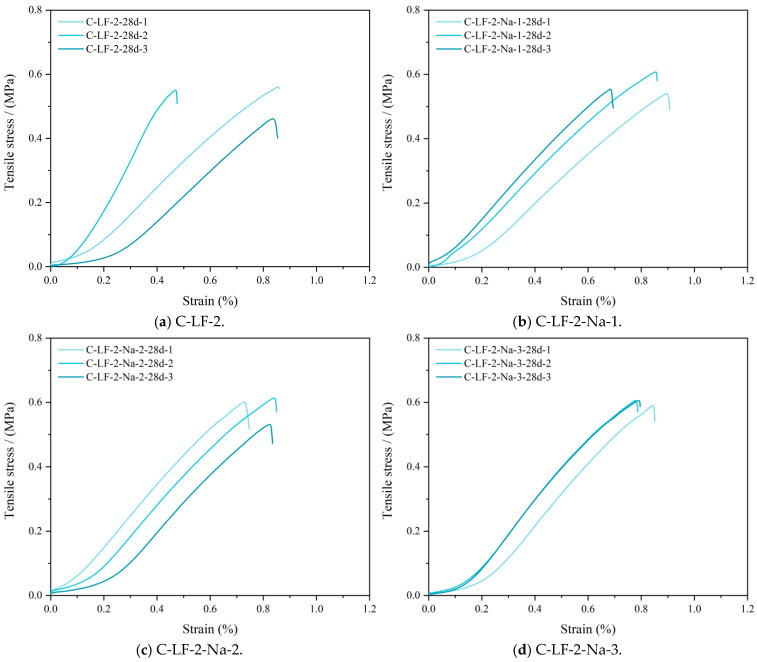
The tensile stress–strain curves of cement-stabilized soil with LF and Na_2_SO_4_.

The tensile stress–strain curves of cement-stabilized soil incorporating LF and Na_2_SO_4_ are presented in Figure 15. The observed increase in splitting tensile strain is primarily attributed to the synergistic effect of fiber reinforcement and chemical activation. LFs enhance the deformation capacity by bridging cracks and dissipating energy during tensile loading. Simultaneously, Na_2_SO_4_ acts as a chemical activator, promoting the formation of additional C-S-H/C-A-S-H gels [49], which results in a denser matrix and improved fiber–matrix inter-facial bonding. This combined effect enhances deformation compatibility and delays crack propagation, thereby significantly improving the strain tolerance and tensile ductility of the stabilized soil.

### 3.4. Capillary Water Absorption Characteristics

**Figure 16:**
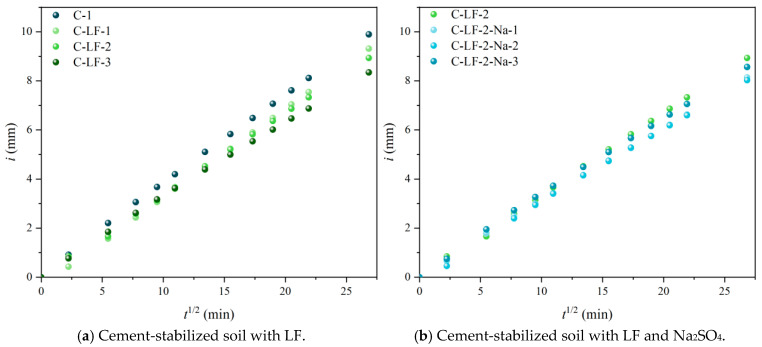
The capillary water absorption of cement-stabilized soil.

The capillary water absorption of cement-stabilized soil is presented in Figure 16. As the LF content increases, a notable reduction in capillary water absorption is observed. This is primarily attributed to the physical modification of the pore structure by the fibers. Dispersed LF fills micro-pores and micro-cracks within the matrix, obstructing capillary channels and extending the water transport path. Additionally, the fiber-induced three-dimensional network increases matrix compactness and reduces interconnected porosity, thereby suppressing capillary action. When Na_2_SO_4_ is incorporated with LF, the absorption rate further declines due to a synergistic physical–chemical enhancement. Na_2_SO_4_ accelerates cement hydration and stimulates pozzolanic reactions, generating more C-S-H and C-A-S-H gels that refine the pore structure and reduce harmful porosity. These gel products intertwine with the fiber network, improving pore filling and forming a denser micro-structural barrier. However, excessive Na_2_SO_4_ may lead to crystallization expansion or accumulation of reaction by-products due to high ion concentration, potentially increasing pore connectivity and weakening the inhibitory effect on water absorption, thus suggesting the presence of an optimal dosage.

### 3.5. Phase Analysis

**Figure 17:**
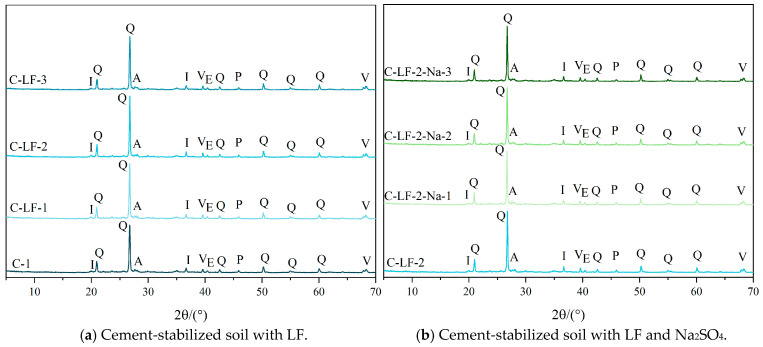
The X-ray diffraction of cement-stabilized soil (Q: quartz, E: ettringite, A: albite, I: illite, P: pyroxene, V: vermiculite).

Figure 17 presents the X-ray diffraction patterns of cement-stabilized soil after 28 days of curing. The primary crystalline phases identified include quartz, ettringite (AFt), albite, illite, pyroxene, and vermiculite. Upon the addition of cement and lime, distinct diffraction peaks corresponding to AFt appear, indicating that cement and lime react with the active SiO_2_ and Al_2_O_3_ present in the soil to generate hydration products such as C-S-H, C-A-S-H, and AFt. These products effectively bind soil particles and contribute to the strength development of the stabilized soil. However, due to the amorphous nature and lack of long-range crystalline order of C-S-H and C-A-S-H gels, their presence is not readily detectable by XRD [50,51]. Their formation is instead confirmed by the SEM observations shown in Figure 5c. Additionally, the incorporation of Na_2_SO_4_ further enhances the intensity of the AFt diffraction peaks. This enhancement is attributed to the role of Na_2_SO_4_ in accelerating cement hydration, particularly by promoting the reaction between tricalcium aluminate (C_3_A) and sulfate ions ($SO_{4}^{2-}$) to form more Aft [52]. Furthermore, Na_2_SO_4_ acts as a dispersing agent, refining the early age capillary pore structure and providing additional nucleation sites for AFt, thus intensifying its XRD signal.

### 3.6. Micro-Structure Analysis

**Figure 18:**
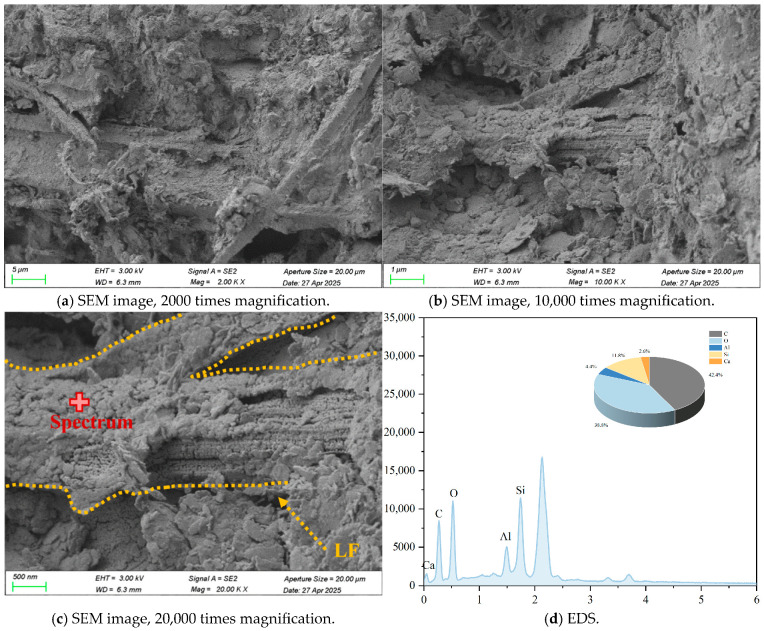
Typical SEM images and EDS elemental analysis of C-LF-2 samples at 28 days.

**Figure 19:**
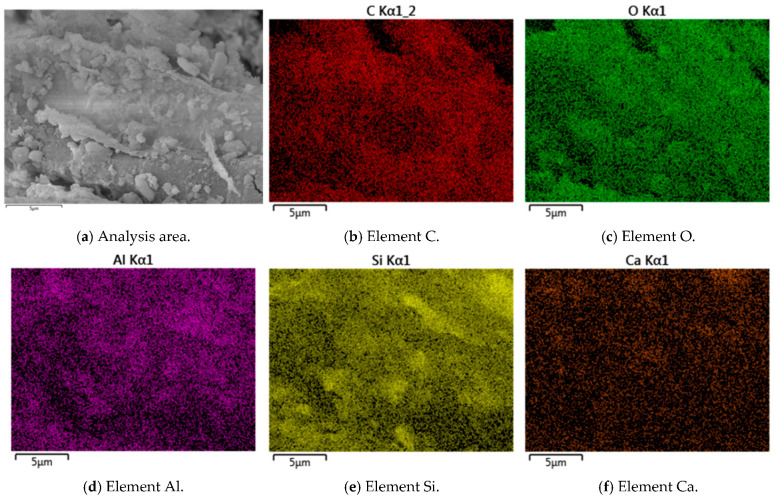
Back-scattering surface scanning analysis of C-LF-2 samples at 28 days.

**Figure 20:**
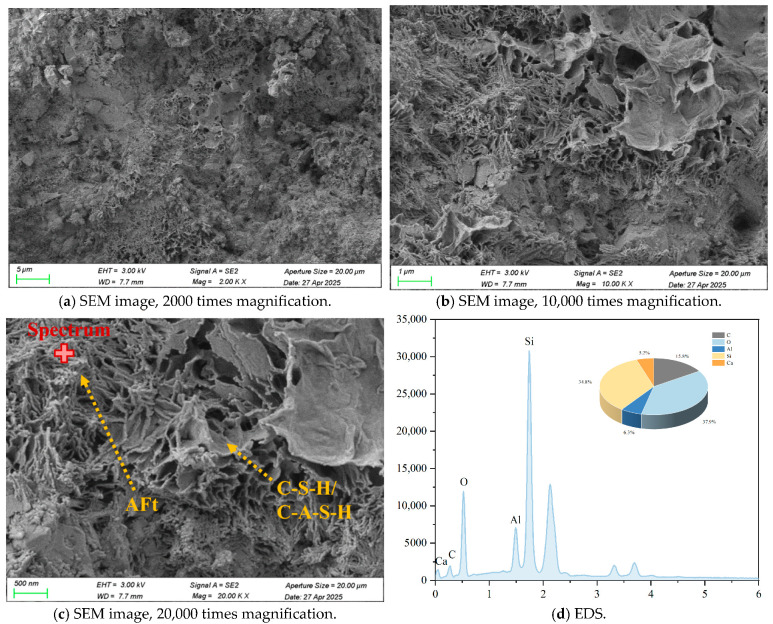
Typical SEM images and EDS elemental analysis of C-LF-2-Na-2 samples at 28 days.

**Figure 21:**
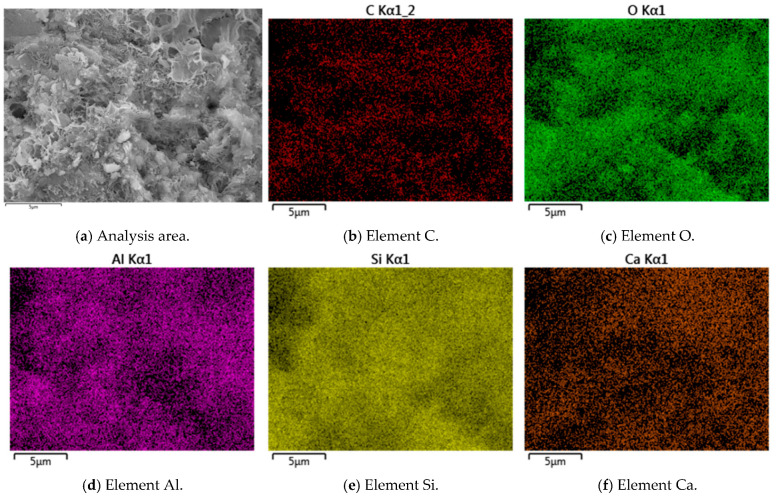
Back-scattering surface scanning analysis of C-LF-2-Na-2 samples at 28 days.

The micro-structure of cement-stabilized soil was investigated to evaluate the effects of LF and Na_2_SO_4_ on soil solidification. Representative SEM, EDS, and back-scattered electron (BSE) images of C-LF-2 and C-LF-2-Na-2 samples at 28 days are shown in Figure 18, Figure 19, Figure 20 and Figure 21. As observed, LFs bridge adjacent soil particles and exhibit a tight inter-facial bond with the cementitious matrix. Numerous fine soil particles and hydration products adhere to the fiber surfaces, contributing to improved mechanical properties. The addition of Na_2_SO_4_ further promotes the formation of C-S-H and C-A-S-H gels, which fill inter-particle voids and enhance the micro-structural integrity. Furthermore, sulfate ions promote the crystallization of sulfate minerals (CaSO_4_·2H_2_O) and stimulate the generation of needle-like AFt during the later stages of hydration [53]. These expansive hydration products fill pores and interlock soil particles, forming a skeletal framework that, in synergy with gel products, results in a denser and mechanically stronger cemented soil structure.

**Figure 22:**
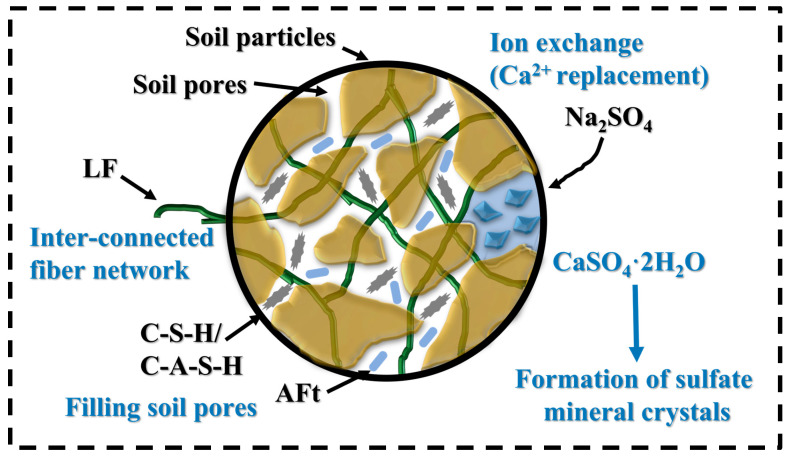
Schematic diagram of the mechanism of cement-stabilized soil with LF and Na_2_SO_4_.

The enhancement in strength and toughness can be attributed to the combined effects of mechanical reinforcement and micro-structural refinement, as demonstrated by both mechanical testing and micro-structural observations. As illustrated in Figure 22, LF and Na_2_SO_4_ exhibit a synergistic physical and chemical reinforcement mechanism. LF provides a physical binding effect by forming a three-dimensional interconnected fiber network that bridges soil particles, promotes aggregate formation, improves pore structure, enhances cohesion, and reduces porosity. Simultaneously, Na_2_SO_4_ serves as a chemical activator, accelerating cement hydration and stimulating pozzolanic reactions. This results in the generation of additional hydration products, including C-S-H, C-A-S-H, and AFt, which effectively fill pores, refine the micro-structure, and increase matrix density and integrity. Furthermore, Na_2_SO_4_ promotes ion exchange processes (e.g., Ca^2+^ substitution) and facilitates the crystallization of sulfate minerals such as gypsum (CaSO_4_·2H_2_O), contributing further to pore filling and matrix densification. This dual mechanism enhances the inter-facial bonding between LFs and clay minerals and promotes the formation of a stable spatial network structure, thereby improving stress distribution, suppressing particle slippage and crack propagation, increasing load-bearing capacity, and reducing soil permeability.

## 4. Conclusions

This study proposes a sustainable stabilization strategy for cement-stabilized soil by incorporating bio-based LF and low-cost Na_2_SO_4_, both of which are derived from industrial by-products. The combined use of these additives aims to enhance the mechanical properties and micro-structural characteristics of stabilized soil. A comprehensive investigation was conducted through a series of macro-scale mechanical tests and micro-structural characterizations. The key findings are summarized as follows:

(1) Optimal synergistic ratio and performance enhancement: When 1.0% LF and 0.10% Na_2_SO_4_ were simultaneously incorporated, the cement-stabilized soil exhibited optimal performance enhancement. Compared with unmodified cement-stabilized soil, the unconfined compressive strength increased by 9.23%, and the splitting tensile strength improved by 18.37%. This optimal ratio also accelerated early strength development and enhanced load-bearing capacity, demonstrating the effectiveness of the synergistic reinforcement.

(2) Improvement in water resistance: The composite modification significantly reduced the capillary water absorption of the stabilized soil. LF formed a physical fiber network that bridged micro-cracks, extended the water transport path, and reduced pore connectivity. Na_2_SO_4_ further refined the pore structure and reduced the proportion of harmful pores by promoting the formation of additional hydration products. Under the optimal dosage, the synergy between physical obstruction and chemical densification provided effective resistance to water penetration.

(3) Micro-structural enhancement mechanism: Micro-structural analyses (SEM, EDS, BSE, and XRD) confirmed the dual-function reinforcement mechanism. LF bridged soil particles and formed tight inter-facial bonds with cement hydration products. Concurrently, Na_2_SO_4_ accelerated the formation and crystallization of key hydration products, including C-S-H, C-A-S-H gels, AFt, and CaSO_4_·2H_2_O. These products interlaced with the LF network, filling inter-particle voids, blocking capillary pores, and enhancing particle bonding, resulting in a denser matrix with reduced porosity and improved mechanical performance.

In conclusion, this study demonstrates the feasibility and efficacy of using industrial by-products such as LF and Na_2_SO_4_ as sustainable modifiers for cement-stabilized soil. The proposed approach enhances compressive and tensile strength, toughness, and water resistance, while reducing dependence on conventional high-carbon cementitious materials. This method offers substantial environmental and engineering value, particularly for roadbed reinforcement and other infrastructure applications. However, its application is affected by the scale of application and the variability of raw materials. Further research and verification are needed in the future.

## Figures and Tables

**Table 1:** Basic physical characteristics of soil used in this study.

Maximum Dry Density (g/cm^3^)	Optimal Moisture Content (%)	Liquid Limit (%)	Plastic Limit (%)	Plasticity Index	Properties of Soil
1.67	17.94	30.4	16.3	14.1	silty clay

**Table 2:** The experimental design scheme of mixed proportions used in this study.

Mix ID	Matrix		Curing Agent		Reinforcing Agent	
	Soil	Water	Cement	Lime	LF	Na_2_SO_4_
R-0	1	0.1794	0	0	0	0
C-1	0.93	0.1794	0.525	0.175	0	0
C-LF-1	0.93	0.1794	0.525	0.175	0.005	0
C-LF-2	0.93	0.1794	0.525	0.175	0.01	0
C-LF-3	0.93	0.1794	0.525	0.175	0.015	0
C-LF-2-Na-1	0.93	0.1794	0.525	0.175	0.01	0.0005
C-LF-2-Na-2	0.93	0.1794	0.525	0.175	0.01	0.001
C-LF-2-Na-3	0.93	0.1794	0.525	0.175	0.01	0.0015

## Data Availability

The original contributions presented in the study are included in the article. Further inquiries can be directed to the corresponding author.
